# The political determinants of the health of undocumented immigrants: a comparative analysis of mortality patterns in Switzerland

**DOI:** 10.1186/s12889-022-13188-8

**Published:** 2022-04-22

**Authors:** Lorenzo Piccoli, Philippe Wanner

**Affiliations:** 1 European University Institute, Robert Schuman Centre for Advanced Studies, Via Giovanni Boccaccio 121, 50133 Florence, Italy; 2 University of Geneva, Institute of Demography and Socioeconomics, 24 rue du Général-Dufour, 1211 Geneva, Switzerland

**Keywords:** Undocumented immigrants, Access to health care, Cause of death, Mortality, Citizenship, Civic stratification, Irregular immigrants, Migration

## Abstract

**Background:**

The health of undocumented immigrants is an important concern in most societies. However, there is no conclusive evidence that inclusive health care policies lead to better outcomes for this group of the population. The aim of this study is to analyse whether there is an association between inclusive health care policies and the mortality patterns of undocumented immigrants, or the distribution of different causes of death among those who have died.

**Methods:**

We analyse individual data concerning the deceased in Switzerland between 2011 and 2017. We proceed in two steps. First, we estimate and compare the patterns of mortality of Swiss citizens, documented immigrants, and undocumented immigrants. Second, we test whether there is an association between cantonal authorities’ policies and differing mortality patterns. We use logistic regressions and multinomial regressions to estimate the relationship between legal status and mortality patterns both in Switzerland and across different cantons.

**Results:**

We find a difference in the patterns of mortality between undocumented immigrants and the other groups of the population. Specifically, death from circulatory system diseases is twice as frequent among undocumented immigrants compared to documented immigrants and Swiss citizens. However, this difference is smaller in the Swiss cantons that have more inclusive health care policies towards undocumented immigrants.

**Conclusions:**

We interpret these results as an indication that policies that expand access to health services lead to better outcomes for undocumented immigrants. This finding has implications for research on civic stratification and public health. Further analysis is needed to evaluate the effects of extending public health care for undocumented immigrants in different contexts.

**Supplementary Information:**

The online version contains supplementary material available at 10.1186/s12889-022-13188-8.

## Background

The health of undocumented immigrants represents an important concern in most societies [1–6]. Yet, this group of the population is often excluded from public benefits. Lying at the very bottom of the structure of civic stratification [7, 8], undocumented immigrants generally lack the full set of heath care rights and entitlements enjoyed by citizens and legally present residents. Previous studies have shown that the legal, social, and material barriers to health care that undocumented immigrants face differ based on the policies enacted by national and subnational governments [9–12], but have not provided conclusive empirical evidence that inclusive health care policies lead to better outcomes for undocumented immigrants.

The question of whether there is an association between inclusive health care policies towards undocumented immigrants and their health is especially crucial against the backdrop of the COVID-19 pandemic. Early studies on the pandemic show that this group of the population is exposed to specific vulnerabilities due to precarious living conditions [13, 14], over-representation in low-skilled professions [15], and fear to be reported to the authorities upon using health care services [16, 17]. In this context, it is important to examine whether inclusive health care policies help tackling these vulnerabilities.

Theoretically, we can imagine that inclusive health care policies protect undocumented immigrants’ health in many ways. For example, public authorities may formally extend health care coverage to undocumented immigrants and promote more accessible insurance coverage [10]. Lower financial barriers to health insurance and greater access to hospitals and doctors can further facilitate inclusion into the public health care system [18, 19]. Dedicated outreach activities may convince undocumented immigrants to seek health care by moderating potential problems related to income disparity, fear of deportation, ﻿lack of familiarity with the local health care system, shortage of providers who speak their native language, and discriminatory care and treatment by health practitioners [20]. By reducing the barriers to health for undocumented immigrants, we expect inclusive policies to be conducive to more similar patterns of mortality – namely, the distribution of different causes of death among those who have died – for citizens and undocumented immigrants.

In this article, we document the patterns of mortality of undocumented immigrants by comparing the distribution of deaths to those of documented immigrants and Swiss citizens. Then, we study the association between the patterns of mortality of undocumented immigrants and the underlying policies of different subnational governments in Switzerland. Due to the considerable autonomy they have in designing health care policies, Swiss cantons can set different priorities regarding the organization and implementation of health care in their territory [21]. Focusing on the Swiss case, we can therefore test whether there is an association between the cantonal authorities’ differing policies and mortality patterns, while controlling for a series of factors that might be endogenous to the country [22].

We use individual mortality data in the 7 years between 2011 and 2017 to answer two questions. The first question is: Do the mortality patterns of undocumented immigrants differ from those of Swiss citizens and documented immigrants? As a second step, we ask: Is there an association between more inclusive cantonal health care policies towards undocumented immigrants and their patterns of mortality?

### Study context

Many of the recently arrived immigrants in Europe live without authorisation. The most recent studies on the subject have estimated the total number of undocumented immigrants living in Europe at around four million [23, 24] and around 80,000 in Switzerland [25].

Undocumented immigrants in Europe generally have access to emergency health care services. However, higher levels of care – such as access to screening and preventive treatment for any infectious disease – are accorded only in some states or in relation to specific situations [26]. In Switzerland, documented and undocumented immigrants have traditionally enjoyed the right to emergency health care without insurance; for all other health services that are not considered urgent, undocumented immigrants must be insured or pay out of their pockets. The 1996 Federal Law on Health Insurance (‘Loi fédérale sur l’assurance-maladie’) requires public insurance companies to accept all persons irrespective of their legal and health status. However, few undocumented immigrants can actually obtain health insurance because of high costs or fear to be reported to the police [27].

Against this common framework, cantons can affect the availability and accessibility of health care rights for the entire population [21] and undocumented immigrants in particular [28, 29]. They can, for example, steer priorities in disease prevention and health education. Furthermore, they operate public hospitals and subsidise private institutions. Cantonal authorities can decide to financially subsidise those individuals whose basic resources are insufficient to cover the cost of social insurance. Finally, cantons can supplement the lack of insurance by creating alternative access channels that function without requiring health care insurance.

## Methods

### Study population: deceased individuals in Switzerland between 2011 and 2017

We focus on the population of individuals who have died on Swiss territory. Mortality data was provided by the Swiss Federal Statistical Office. The file we received contains records of all the deaths registered with the office of Civil Registration between 2011 and 2017, with information on the age, place of death, residence status, sex, and nationality. The quality of cause-of-death declarations in Switzerland is comparable to other high-income countries, and generally better in terms of the proportion of ‘Insufficiently specified’ codes [30]. We expect the quality of reporting for different groups of the population to be consistent because physicians certify death regardless of the domicile status of the deceased. The number of observations for the 2011–2017 period is *n* = 457,780. An overview of our data can be found in the Supplement Materials.

We want to identify the population of deceased undocumented immigrants. As a first step, we exclude individuals with documented status: Swiss citizens and foreigners regularly residing (permanently or not permanently) on Swiss territory. As a second step, we exclude tourists and patients coming to Switzerland to receive care in Swiss clinics and hospitals, especially in those cities located on the border with Germany (Basel) and France (Geneva). We therefore exclude citizens of the EU and the EFTA because they are covered by free circulation agreements and have regular access to the Swiss territory.

Following previous models [31, 32], we then exclude individuals from countries classified by the WHO as group A (Australia, Brunei, Cuba, Canada, Israel, Japan, New Zealand, Singapore, United States) and from three small territories in Europe (Andorra, Monaco, San Marino). These countries generally do not generate flows of undocumented immigrants to Switzerland. Finally, we remove: 78 observations in which the nationality of the persons is not known; 37 observations whose cause of death was not documented; and 97 observations concerning citizens of foreign countries not belonging to group A holding a permit of residence in an EU/EFTA country (e.g., Turkish citizens residing in Germany; Latin American citizens residing in France). We are left with 679 observations, who are likely to have had undocumented residence status in Switzerland at the time of death.

### Observed variables: nationality, sex, age, cause of death, and canton of death

We analyse available information on nationality, sex, age, underlying cause of death, and canton of death. We could not analyse socio-economic variables, such as the duration of stay in Switzerland and education level, because they were not included in the data we received.

### Independent variables: political determinants

We focus on cantonal policies as explanatory factors. We hypothesise that more inclusive health care policies towards undocumented immigrants will be associated with more consistent mortality patterns vis-à-vis other groups in the population (i.e., Swiss citizens and regular immigrants). Based on existing studies on Swiss cantonal health care policies towards undocumented immigrants, we distinguish between the different “health landscape[s] that [undocumented] immigrants must navigate” [2]. We therefore divide Swiss cantons into three groups, which we summarise in Table 1.

**Table 1:** Groups of cantons divided per type of health care policies towards undocumented immigrants

Label	Type	Cantons (with population in 2020)	Notable urban areas (with population in 2020)	Swiss population living in these cantons	Deaths of undocumented immigrants: 2011–2017
Group 1: Inclusive Policies	Inclusion of undocumented immigrants in the mainstream system	Geneva (506,343), Vaud (814,762)	Geneva (203,856), Lausanne (140,202)	15% (1,321,105)	33.4% (227)
Group 2: Fragmented Policies	No structured policy, but coordination with NGOs with public funding	Basel-Stadt (201,156), Fribourg (325,496), Lucerne (416,347), Jura (73,709), Neuchatel (175,894), Ticino (350,986), Zürich (1,553,423)	Basel (178,120), Lucerne (82,620), Zürich (421,878), Winterthur (114,220)	36% (3,097,011)	40.5% (275)
Group 3: No Policy	No public policy in place	Aargau (694,072), Appenzell Innerrhoden (16,293), Appenzell Ausserrhoden (55,309), Basel-Land (292,955), Bern (1,043,132), Glarus (40,851), Grisons (200,096), Nidwalden (43,520), Obwalden (38,108), San Gallen (514,504), Schaffhausen (83,107), Schwyz (162,157), Solothurn (277,462), Thurgau (282,909), Uri (36,819), Valais (348,503), Zug (128,794)	Bern (134,794), Biel (55,206)	49% (4,258,591)	26.7% (177)

*Note*:Own elaboration based on [27, 28]

Geneva and Vaud belong to the group of cantons with inclusive health care policies. These cantons provide health services to undocumented immigrants within the public health care system and complement it with other services that encompass food and shelter provided by NGOs. They also apply a low-threshold and, in Geneva, an outreach approach. Seven other cantons do not have a structured policy towards undocumented immigrants but coordinate action and provide limited funding to different NGOs. They generally have small structures with no comprehensive care towards undocumented immigrants. If the needs are complex, individuals can however be helped to obtain health insurance – e.g., free health in Fribourg. Finally, 17 cantons do not have dedicated services and do not provide funding to NGOs, although individual hospitals can negotiate with the public institutions to apply a lower tariff or to authorise payment by instalments.

The three groups are generally comparable on important parameters other than the health care policy towards undocumented immigrants. Each group contains at least one canton on Switzerland’s borders (Geneva, Ticino, San Gallen); a combination of cities (Geneva, Zürich, Bern) and rural areas (Vaud, Jura, Appenzell Innerrhoden); and a combination of high-tourism density (Geneva, Luzern, Valais) and low-tourism density (Vaud, Neuchatel, Schaffhausen). Due to the different size of the groups, there are some differences that we discuss in the section on Strengths and Limitations: in particular, the cantons of Geneva and Vaud are generally more densely populated and have a younger population than cantons in the other groups. In the Supplement Materials we provide additional information to better evaluate the comparability across the three groups of cantons, including group-specific information on the patterns of mortality, the distribution of deaths according to age, and the distribution of nationality among deceased undocumented immigrants.

### Statistical analysis

We analyse mortality patterns according to the cause of death. We run logistic regressions [33] to measure the statistical association between legal status (undocumented immigrants versus Swiss citizens and documented immigrants) and the cause of death (coded according to the ICD-10: infectious diseases caused by pathogenic microorganisms; neoplasms; cardiovascular diseases resulting from conditions affecting the heart or blood vessels; external causes due to accidents and violence including suicide, fall, poisoning, and other adverse effects; and all other causes, including respiratory and liver diseases). This approach is based on previous research conducted in Belgium [32] and Sweden [31]. Expanding on these studies, we also analyse the proportion of mortality that can be avoided: preventable mortality – defined as deaths that could be avoided through public health and prevention interventions – and amenable mortality – defined as deaths that could be avoided through effective and timely health care. We distinguish these two types of mortality using the OECD/Eurostat standards as reference definitions [34].

The regressions aim to explain the probability (expressed as an odds ratio, p) of dying from one cause, compared to other causes according to legal status. The formula is as follows:

$$\mathrm{logit}\left(\mathrm{p}\right)=\ln \kern0.5em \left(\mathrm{p}/\left(1-\mathrm{p}\right)\right)={\upbeta}_0+{\upbeta}_1\ {\mathrm{x}}_{\left(\mathrm{i},1\right)}+{\upbeta}_2\ {\mathrm{x}}_{\left(\mathrm{i},2\right)}+\cdots$$

With β_0_ being a constant and β_(1,…n)_ being the coefficients of the explanatory variables x__(1,… n)_, and the exponential value of β_(1,…n)_ being the odds ratios. For all models, we present the levels of significance (* *p* < 0.05; ** *p* < 0.01; ***p < 0.001) to facilitate the interpretation of the results. Apart from the population’s legal status, we include age in the models as a control variable in nine groups (< 20, 20–29, 30–39, 40–49, 50–59, 60–69, 70–79, 80–89, 90+). This is based on the observation that the demographic structure of the population of undocumented immigrants living in Switzerland is estimated to be significantly younger compared to the population of Swiss citizens and documented immigrants [25, 35]. The second range of models includes the region where the deceased person holds nationality (Europe, Africa, Latin America, Asia). We also run a broad range of models for the five groups of causes of deaths, comparing undocumented immigrants with Swiss citizens, all documented immigrants, and all immigrants of the same origin. Then we run the models for the three groups of cantons, separately.

Finally, we use a multinomial logistic regression to examine the joint effects of different variables. We fit two predictor variables: the group of cantons (Group 1: Inclusive Policies; Group 2: Fragmented Policies; Group 3: No Policy) and legal status (undocumented versus Swiss). The outcome variable is the cause of death (infections, neoplasms, cardiovascular diseases, and external causes compared to death for other reasons). We include three models: Model 1 without interactions, adjusted for age; Model 2 with interactions, not adjusted for age, nationality, and sex; and Model 3 with interactions, adjusted for age, nationality, and sex.

## Results

### Descriptive statistics of the deceased population

Table 2 allows a preliminary comparison between undocumented immigrants, documented immigrants, and Swiss citizens. About 60% of the population of deceased undocumented individuals are men, the majority of whom come from Asia and Balkan countries. The mean age of death is significantly lower for men (51) than for women (59). The average age of death of undocumented male immigrants is lowest for those coming from Sub-Saharan African countries (41), while for undocumented female immigrants it is lowest among those coming from the Maghreb (48).

**Table 2:** Size and age of death of the deceased population per status, nationality and sex

	Nationality	Men			Women		
		N	Age of death		N	Age of death	
			Mean	Std		Mean	Std
Undocumented immigrants	Balkan countries	119	52	21	69	67	17
	Other Europe	62	50	21	49	60	18
	The Maghreb	47	42	23	19	48	31
	Other Africa	48	41	22	29	53	22
	Latin America	29	44	24	28	49	25
	Asia	123	58	21	57	60	21
	All	428	51	22	251	59	22
Swiss citizens		188,063	77	15	213,787	82	14
Documented immigrants		24,694	69	18	15,905	74	20

Source: FSO

*Note*:Deceased undocumented immigrants represent a total of 85 different nationalities

The comparison between the different groups highlights some apparent differences. The mean age of death of undocumented immigrants is significantly lower (mean ages at death: 54 for both sexes; 51 for men; 59 for women) than documented immigrants (71 for both sexes; 69 for men; 74 for women), and Swiss citizens (80 for both sexes; 77 for men; 82 for women). The proportion of deaths for age cohort within each group of the population, which we include in the Supplement Materials, shows that the mortality of the undocumented population is significantly higher than the mortality of the Swiss population in four age cohorts: 0–4 (7.4% of deaths among undocumented immigrants versus 3.3% of deaths among Swiss citizens) and then again in the age cohorts 25–29 (9.3% versus 1.8%), 30–34 (9.8% versus 1.8%), and 34–39 (8.3% versus 2.7%). These differences deserve further research. Theoretically, they could be partially explained by the different age structure of the two groups of the population: for example, if the population of undocumented immigrants is younger than the population of Swiss, they will likely give birth to relatively more children, which in turn will lead to higher infant mortality compared to the other groups of the population.

**Fig. 1:**
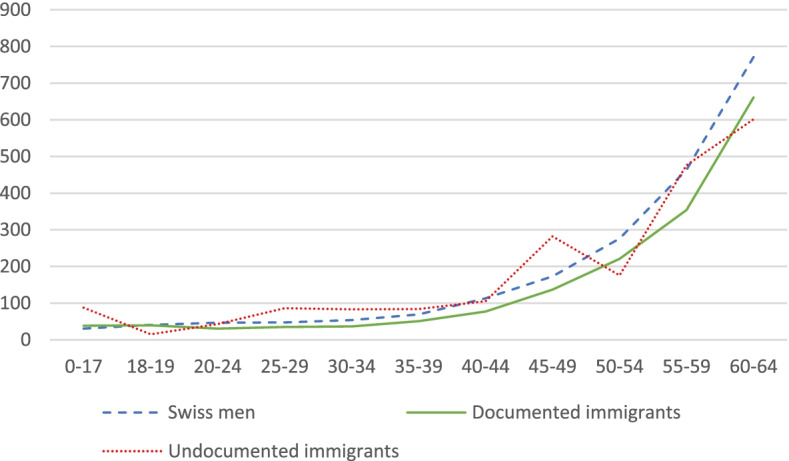
Estimated mortality rate by age for Swiss men, documented male immigrants, and undocumented male immigrants Note: Own elaboration based on data from FSO and (25)

**Fig. 2:**
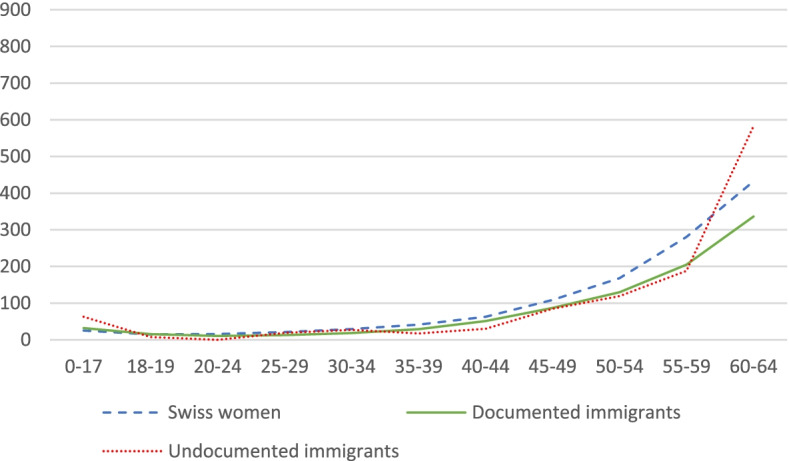
Estimated mortality rate by age for Swiss women, documented female immigrants, and undocumented female immigrants. Note: Own elaboration based on data from FSO and (25)

Regrettably, we do not have precise information on the age structure of undocumented immigrants in Switzerland; but we know from previous research that this group of the population is generally considered to be younger than the rest of the population [25, 35], as it is the case also in other countries outside of Europe [36]. We use existing estimations of the age structure of undocumented immigrants to investigate mortality rates [25]. We distribute the undocumented population in Switzerland by age and sex, estimate a smoothed age profile, and calculate annual mortality rates by sex and age (per 100,000). We limit the analysis to individuals aged 0–64, based on the assumption that the size of the undocumented population aged more than 65 is negligible. We illustrate the results in Fig. 1 and Fig. 2. Overall, undocumented men have a mortality rate that is 9% higher compared to Swiss men, while undocumented women have a mortality rate that is 1.3% higher than Swiss women.

### Cause of death

Moving to the patterns of mortality in Table 3, we observe that cardiovascular diseases are the most frequent of the first four causes of death for all groups analysed. For undocumented immigrants, they constitute about 30% of the deaths of both men and women, which is more than the proportion observed among documented immigrants (approximately 25%). The group of male undocumented immigrants stands out in terms of death from external causes with a frequency of 25%, compared to 16% for documented immigrants and 8% for Swiss citizens. By contrast, neoplasm is a less frequent cause of death among male undocumented immigrants (17%) than it is for documented immigrants (34% and for Swiss citizens (28%). Finally, while infectious diseases are rarely observed, they are more frequent among undocumented citizens (2% for men and women) than they are for documented immigrants and Swiss citizens (1% for men and women).

**Table 3:** Distribution of the causes of death according to status and sex

	Undocumented immigrants				Documented immigrants				Swiss citizens			
	Men		Women		Men		Women		Men		Women	
	N	%	N	%	N	%	N	%	N	%	N	%
Infectious diseases	9	2.1	6	2.4	345	1.4	229	1.4	2044	1.1	2299	1.1
Neoplasms	72	16.8	62	24.7	8510	33.7	4856	29.8	53,359	28.4	45,700	21.4
Cardiovascular diseases	124	29.0	75	29.9	6010	23.8	4242	26.1	57,114	30.4	72,368	33.9
External causes	106	24.8	23	9.2	3970	15.7	1708	10.5	14,199	7.6	11,629	5.4
Other causes	117	27.3	85	33.9	8182	32.4	6011	36.9	64,454	34.3	85,789	40.1
All causes	428	100.0	251	100.0	25,239	100.0	16,276	100.0	188,061	100.0	213,787	100.0

Source: FSO

Next, we compare age-adjusted differences in patterns of death between undocumented immigrants and Swiss citizens, undocumented immigrants and documented immigrants, and undocumented immigrants and documented immigrants of the same nationality, stratified by sex. Table 4 should be interpreted with prudence: it does not include interaction effects between legal status and cause of death. The results show that dying for diseases of the circulatory system is significantly more frequent among undocumented men compared to Swiss citizens (OR 2.0, CI: 1.6–2.4, *p* < 0.001) and undocumented women (OR 2.4, CI: 1.8–3.1, *p* < 0.001). Also, death from infectious diseases is more frequent among undocumented men (OR 2.1, CI: 1.2–3.7, *p* < 0.05) and undocumented women (OR 2.3, CI: 1.1–4.7, *p* < 0.05). Conversely, death caused by neoplasms is less frequent (OR 0.5, CI: 0.4–0.7, *p* < 0.001 for both men and women).

**Table 4:** Odds ratios of mortality for different causes of undocumented immigrants compared to Swiss citizens, documented immigrants, and documented immigrants of the same origin, adjusted for age

	Undocumented immigrants versus Swiss citizens					
	Men			Women		
	OR	95% CI	*P*	OR	95% CI	*P*
Infectious diseases	2.1	1.2–3.7	*	2.3	1.1–4.7	*
Neoplasms	0.5	0.4–0.7	***	0.5	0.4–0.7	***
Cardiovascular diseases	2.0	1.6–2.4	***	2.4	1.8–3.1	***
External causes	1.0	0.8–1.3		0.8	0.5–1.2	
Other causes	0.9	0.7–1.1		1.0	0.7–1.2	
	Undocumented immigrants versus documented immigrants					
	Men			Women		
	OR	95% CI	*P*	OR	95% CI	*P*
Infectious diseases	1.7	1.0–3.1		1.8	0.9–3.7	
Neoplasms	0.5	0.4–0.7	***	0.6	0.4–0.8	***
Cardiovascular diseases	2.0	1.6–2.4	***	2.3	1.8–3.0	***
External causes	1.0	0.8–1.3		0.6	0.4–0.8	**
Other causes	0.9	0.7–1.1		1.1	0.9–1.4	
	Undocumented versus documented immigrants of the same origin					
	Men			Women		
	OR	95% CI	*P*	OR	95% CI	*P*
Infectious diseases	1.6	0.9–3.1		1.0	0.5–2.1	
Neoplasms	0.4	0.3–0.6	***	0.5	0.4–0.7	***
Cardiovascular diseases	1.7	1.4–2.2	***	1.9	1.4–2.5	***
External causes	1.9	1.4–2.4	***	1.6	1.1–2.5	**
Other causes	0.8	0.6–1.0	*	0.9	0.7–1.2	

Source: Own elaboration, based on data from FSO. Results of logistic regressions after taking into account the age group. * *p* < 0.05 ** *p* < 0.01 *** *p* < 0.001

The difference in the patterns of mortality between undocumented immigrants and documented immigrants is similar to the difference in the patterns of mortality between undocumented immigrants and Swiss citizens (second part of Table 4). For men, for example, death caused by diseases of the circulatory system is 2.3 times higher for undocumented immigrants than it is for documented immigrants (CI: 1.7–3.1, *p* < 0.001), while mortality caused by neoplasm is 60% lower (OR 0.4, CI: 0.3–0.6, *p* < 0.001).

We obtain similar results comparing the patterns of mortality of undocumented immigrants and documented immigrants of the same origin (third part of Table 4). For undocumented women, for example, death caused by cardiovascular diseases is 1.9 times higher than it is for documented women of the same origin (CI: 1.4–2.5, *p* < 0.001). Again, for neoplasms the trend is reversed, with odds ratios of 0.5 (CI: 0.4–0.7, *p* < 0.001).

We introduce additional controls, which we include in the Supplement Materials: logistic regressions with the origin (dummy variable) to control the impact of the behaviours/genetic characteristics on the results. The results are consistent.

We then study the distribution of amenable and preventable mortality among the different groups of the population. The proportion of deaths that can be avoided is higher in the group of undocumented immigrants. Table 5 shows an increase in the frequency of amenable deaths among undocumented men compared to Swiss men (OR 1.6, CI: 1.3–2.0, *p* < 0.001) and undocumented women compared to Swiss women (OR 1.8, CI: 1.4–2.4 *p* < 0.001). There is no clear pattern for preventable deaths. We interpret this result as driven by the fact that preventable deaths include a larger range of malignant neoplasms (liver, lung) as well as external deaths. Higher odds ratios for deaths amenable to treatment highlight the negative consequences of the lack of access to health care for undocumented immigrants, as these deaths could be avoided through more effective health care policies. By contrast, preventable deaths are caused by broader behavioral, socio-economic, environmental, and lifestyle factors, as well as a reduction in the exposure to factors of risk in the workplace and in housing.

**Table 5:** Odds ratios of amenable and preventable deaths of undocumented immigrants compared to Swiss citizens, documented immigrants, and documented immigrants of the same origin

	Undocumented immigrants versus Swiss citizens					
	Men			Women		
	OR	95% CI	*P*	OR	95% CI	*P*
Amenable deaths	1.6	1.3–2.0	***	1.8	1.4–2.4	***
Preventable deaths	1.1	0.9–1.3		1.0	0.8–1.4	
	Undocumented immigrants versus all documented immigrants					
	Men			Women		
	OR	95% CI	*P*	OR	95% CI	*P*
Amenable deaths	1.5	1.2–1.9	***	1.6	1.2–2.1	**
Preventable deaths	1.1	0.9–1.3		1.2	0.9–1.6	
	Undocumented immigrants versus documented immigrants of the same origin					
	Men			Women		
	OR	95% CI	*P*	OR	95% CI	*P*
Amenable deaths	1.4	1.1–1.8	**	1.4	1.0–1.9	*
Preventable deaths	1.2	0.9–1.4		1.3	1.0–1.8	

Source: Own elaboration, based on data from FSO and definitions of Eurostat/OECD. Results of logistic regressions after taking into account the age group. * *p* < 0.05 ** *p* < 0.01 *** *p* < 0.001

### Cantonal differences

Having assessed the patterns of mortality of undocumented immigrants in Switzerland, we measure whether such patterns vary across cantons. Specifically, we test whether the difference in mortality patterns between citizens and undocumented immigrants is less pronounced in cantons with more inclusive health care policies towards undocumented immigrants than it is in cantons with fragmented policies and in cantons with no policy.

**Fig. 3:**
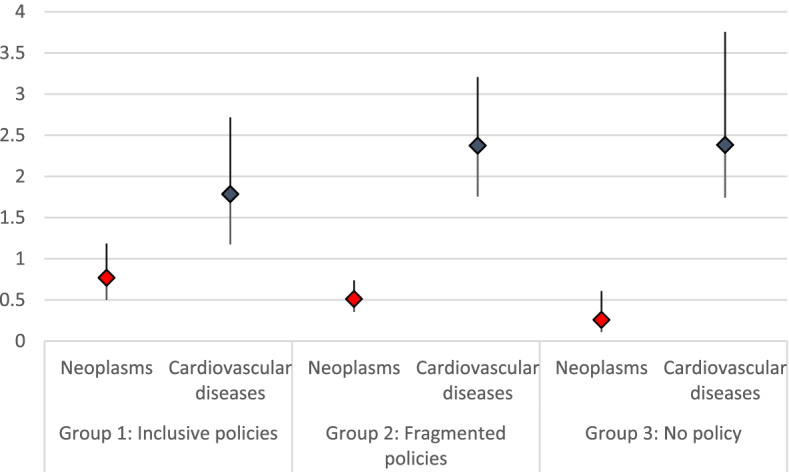
Odds ratios of mortality caused by neoplasms and cardiovascular diseases of undocumented immigrants compared to Swiss citizens in the three groups of cantons, adjusted for age. Note: Authors’ elaboration, based on data from FSO

In Fig. 3 we illustrate the odds ratios of mortality caused by neoplasm and cardiovascular diseases of undocumented immigrants versus Swiss citizens in the three groups of cantons: Group 1 (Inclusive Policies), Group 2 (Fragmented Policies), Group 3 (No Policy). We compare one cause of death that is generally considered amenable (cardiovascular diseases) and one cause of death that, for the most part, is not (neoplasms). This allows us to observe changes in the relative frequency of deaths that, like cardiovascular diseases, can be reduced through timely detection (e.g., availability of screening services) and effective medical intervention (e.g., appropriate treatment, availability of antibiotics); and deaths that, like most neoplasms, can be reduced mainly through behavioural and social interventions (e.g., interventions aimed at decreased smoking, obesity, and alcohol consumption). Deaths that are due to external causes and to other causes can be reduced by a combination of better health care services and improved living conditions. Infectious diseases are generally considered amenable but, due to the small number of observations for infectious diseases, we decided not to include this cause of death in the figure. Additional tables with regressions for all different causes of death are available in the Supplement Materials.

We interpret Fig. 3 with caution because it does not include interaction effects between canton of death, legal status, and cause of death. We note one interesting trend, however: in cantons with inclusive health care policies the mortality patterns of undocumented immigrants are more similar to the mortality patterns of Swiss citizens. For example, the odds ratio of dying from cardiovascular diseases for undocumented male immigrants is higher in cantons with fragmented policies (OR 2.4, CI: 1.8–3.2, *p* < 0.001) and in the cantons with no policy in place (OR 2.4, CI: 1.4–4.1, *p* < 0.01) than it is in cantons with inclusive policies (OR 1.8, CI: 1.2–2.7, *p* < 0.01). The same trend can be observed for female undocumented immigrants and for the comparison between undocumented immigrants of both sexes and documented immigrants of the same nationality. The trend is reversed for neoplasms, which are less frequent causes of death among undocumented immigrants than they are for Swiss citizens, documented immigrants, and documented immigrants of the same nationality. The frequency of mortality from neoplasm of female undocumented immigrants is one fourth than that of female Swiss citizens in the cantons with no policy (OR 0.2. CI: 0.1–0.6, *p* < 0.01), and it is half that of female Swiss citizens in the cantons of the two groups of cantons with policies in place, either inclusive or fragmented (OR 0.5, CI: 0.4–0.8, *p* < 0.01 and OR 0.6, CI: 0.4–1.0, *p* < 0. 1, respectively). As we move from more inclusive cantons to cantons with fragmented policies and to cantons with no policy, the difference in the patterns of mortality becomes larger.

### Multinomial logistic analysis

We use multinomial logistic analysis as an alternative strategy to test the relationship between legal status and cause of death in Switzerland and across different groups of cantons. The results are presented in Table 6. We include three alternative models. The first model does not contain interactions and is adjusted for age. The second model contains interactions and is not adjusted for age, nationality, and sex; the third model contains interactions and is adjusted for age, nationality, and sex. Adjusting for the age of the deceased explains some differences concerning the odds ratios in the different models. For example, not controlling for age leads to a marked increase in the odds ratio of death from external causes compared to other causes between undocumented immigrants and Swiss citizens. After adjustment, the odds ratio is significantly lower. This can be interpreted as follows: violent deaths occur very often at a young age and, even though we do not have exact information on the age structure of this population, we know that a much higher proportion of undocumented immigrants are young compared to Swiss citizens [28].

**Table 6:** Multinomial logistic regression with odds ratios of dying from infections, neoplasms, cardiovascular diseases, and external causes compared to other causes of death

	Infections			Neoplasms			Cardiovascular diseases			External causes		
	O.R.	95% CI	*P*	O.R.	95% CI	*P*	O.R.	95% CI	*P*	O.R.	95% CI	*P*
**Model 1, without interaction, adjusted**												
*** Status***												
Swiss (reference category)	1.00			1.00			1.00					
Undocumented immigrant	0.92	0.29–2.92		0.69	0.49–0.97	*	2.06	1.52–2.78	***	1.06	0.73–1.53	**
*** Group of cantons***												
Group 1 : Inclusive Policies (reference)	1.00			1.00			1.00					
Group 2 : Fragmented Policies	1.22	1.10–1.34	***	1.03	1.01–1.06	*	1.29	1.26–1.32	***	1.22	1.10–1.34	**
Group 3 : No Policy	1.24	1.11–1.38	***	1.06	1.03–1.09	***	1.38	1.35–1.42	***	1.02	0.98–1.07	
**Model 2, with interactions, not adjusted**												
*** Status***												
Swiss (reference category)	1.00			1.00			1.00			1.00		
Undocumented immigrant	0.86	0.21–3.50		0.84	0.60–1.18		0.77	0.55–1.08		1.82	1.20–2.76	**
*** Group of cantons***												
Group 1 : Inclusive Policies (reference)	1.00			1.00			1.00			1.00		
Group 2 : Fragmented Policies	1.22	1.10–1.34	***	1.06	1.04–1.09	***	1.28	1.25–1.32	***	1.05	1.01–1.09	*
Group 3 : No Policy	1.24	1.11–1.38	***	1.10	1.07–1.13	***	1.38	1.34–1.42	***	1.01	0.97–1.06	
*** Interactive term (undocumented immigrant)***												
Group 1 : Inclusive Policies (reference)	1.00			1.00			1.00			1.00		
Group 2 : Fragmented Policies	5.36	1.14–25.26	*	1.78	1.11–2.85	*	2.32	1.48–3.63	***	3.28	1.94–5.55	***
Group 3 : No Policy	3.66	0.58–22.93		0.61	0.28–1.30		1.48	0.82–2.67		2.35	1.19–4.64	*
**Model 3, with interaction, adjusted**												
*** Status***												
Swiss (reference category)	1.00			1.00			1.00			1.00		
Undocumented immigrant	0.13	0.02–0.87	*	0.66	0.40–1.10		1.58	0.97–2.58		0.36	0.20–0.66	**
*** Group of cantons***												
Group 1 : Inclusive Policies (reference)	1.00			1.00			1.00			1.00		
Group 2 : Fragmented Policies	1.20	1.09–1.32	***	1.03	1.00–1.06	*	1.29	1.26–1.32	***	1.05	1.01–1.09	*
Group 3 : No Policy	1.22	1.10–1.36	***	1.05	1.03–1.09	***	1.38	1.34–1.41	***	1.01	0.97–1.06	***
*** Interactive term (undocumented immigrant)***												
Group 1 : Inclusive Policies (reference)	1.00			1.00			1.00			1.00		
Group 2 : Fragmented Policies	9.53	1.92–47.20	**	1.35	0.80–2.27		1.60	0.97–2.65		4.01	2.18–7.36	***
Group 3 : No Policy	7.92	1.17–53.46	*	0.49	0.22–1.11		1.03	0.54–1.98		3.09	1.44–6.65	**

Note: Model 1: Without interactions. Model 2: With interactions, not adjusted. Model 3: With interactions, adjusted with age, country of origin, and sex

This analysis confirms the excess mortality of undocumented immigrants for cardiovascular diseases when we compare this group of the population to Swiss citizens (Upper part of Table 6, Model without interactions). We also observe an excess mortality for external causes. Unlike simple logistic regressions, however, in this model we do not observe an association between undocumented status and death caused by infectious disease.

The cantonal differences in the distribution of deaths of undocumented immigrants vary considerably per cause of mortality. For cardiovascular diseases, for instance, they trend in the same direction as in the previous model – higher odds ratios in cantons with fragmented policies and in cantons with no policy than in cantons with inclusive policies – but they are not statistically significant. By contrast, we observe statistically significant cantonal differences for deaths due to infectious diseases and external causes. The odds ratios of dying from external death compared to dying from other causes are three to four times higher for undocumented immigrants in cantons with fragmented policies (CI: 2.2–7.4, *p* < 0.001) and in cantons with no policy (CI 1.4–6.7, *p* < 0.001) than they are in cantons with inclusive policies. Neoplasms are less frequent causes of death in cantons with inclusive policies than they are in cantons with fragmented policies; but they are more frequent in cantons with inclusive policies than in cantons with no policy in place. Interestingly, the most striking differences in this analysis are those between inclusive cantons and cantons with fragmented policies, while for cantons with no policy in place the odds ratios are smaller and the statistical significance is lower.

## Discussion

The analysis of the causes of mortality shows that deaths caused by cardiovascular diseases are more frequent among undocumented immigrants than they are among Swiss citizens and documented immigrants. In contrast, deaths caused by neoplasms are less frequent among undocumented immigrants. These results are consistent and expand upon previous studies conducted in Belgium [32] and Sweden [31]: there are significant differences in mortality patterns between undocumented immigrants and citizens. Overall, undocumented immigrants die more frequently than Swiss citizens and documented immigrants for diseases that are considered amenable and that could be avoided through timely detection (e.g., availability of screening services) and effective medical intervention (e.g., appropriate treatment, availability of antibiotics).

Our analysis suggests that in Swiss cantons with more inclusive policies towards undocumented immigrants the difference in the mortality patterns is smaller. Using simple regressions, we observe that in cantons with inclusive policies undocumented male mortality from cardiovascular diseases among undocumented immigrants is less than twice as frequent as that of Swiss citizens, whereas in cantons with no policies it is more than twice as frequent. The multinomial analysis confirms that mortality from cardiovascular diseases is more frequent for undocumented immigrants compared to Swiss citizens; but this analysis also shows that the cantonal differences in undocumented immigrants’ mortality from cardiovascular diseases are not statistically significant. By contrast, the odds ratios of undocumented immigrants’ death caused by infectious diseases and death by external causes are significantly higher – and statistically significant – in cantons with fragmented policies and in cantons with no policy than they are in cantons with inclusive policies.

We interpret these results as an indication that public policies that expand access to health care services lead to better outcomes for undocumented immigrants. Inclusive policies improve the availability of timely treatment services and social protection mechanisms; they provide precise information related to symptoms, prevention, control of spread, treatment, and social relief; finally, they make it less likely that health clinic staff and clinicians make arbitrary assumptions about who deserves access to health care [22]. Overall, the difference in the mortality patterns between citizens and undocumented immigrants is smaller in cantons with health services that are more easily accessible regardless of one’s legal status.

### Study strengths and limitations

Statistics on the health and mortality of undocumented immigrants are key to address the vulnerabilities of this population, monitor progress towards the achievement of migration-related targets in the Sustainable Development Goals, and ensure equality in access to health. Regrettably, such data are still scarce. This is due to the apprehensive nature of the phenomenon and the complexity of linking demographic information with indicators of public policy. Our study provides original data to estimate the patterns of mortality and evaluates the importance of policies in shaping these patterns. We exploit the Swiss register of mortality and cantonal variation as study space, but we consider the findings of broad interest beyond the Swiss case. Our findings advance the public health debate on the political determinants of health [38, 39] and the sociological discussion on the effects of the civic stratification of rights [7, 8].

We see four important limitations to our study design. These limitations are driven by the lack of reliable data on the demographic characteristics of the study population. First, the interpretation of the cantonal differences hinges on the assumption that the demographic characteristics of the population of undocumented immigrants are consistent across the three groups of cantons that we have identified. We reduce the risk of immigrants’ self-selection dividing cantons in three groups that are generally comparable, but we note that cantons of Group 1 (Inclusive Policies) and Group 2 (Fragmented Policies) are generally more densely populated areas with greater economic possibilities than cantons of Group 3 (No Policy). Second, because we do not have accurate data on the age-structure of undocumented immigrants across the cantons, we can only investigate the distribution of various causes of death among those who have died. Third, because our dataset covers a limited time-period of 7 years, we have relatively few observations among the deceased undocumented population. Datasets with longer temporal coverage would make it possible to produce more robust statistical associations. Finally, we do not have information on the duration of stay of an individual in a canton prior to their death. For this reason, a person who is severely ill and lives in a canton without inclusive health care policy may travel to another canton to receive treatment and die in that canton. However, this group of the population is likely to be relatively small since all cantons provide emergency health care regardless of legal status.

## Conclusions

Using an original dataset of mortality in Switzerland between 2011 and 2017 (*n* = 457,780), we identified 679 deaths that were likely to have been undocumented immigrants. We analysed their mortality patterns compared to Swiss citizens and documented immigrants. Among the deceased, those who are undocumented are younger than documented immigrants and Swiss citizens. We estimate that undocumented men have a mortality rate 9% higher compared to Swiss men and undocumented women have a mortality rate 1.3% higher than Swiss women. We show that the proportion of undocumented immigrants who die from diseases that are considered amenable is much higher than the proportion of Swiss citizens and documented immigrants. Specifically, we estimate that undocumented immigrants die twice as often as Swiss citizens and documented immigrants from cardiovascular diseases. These numbers must be treated with prudence, but they point to a difference in the patterns of mortality between Swiss citizens and undocumented immigrants. However, the difference in mortality patterns is smaller in the Swiss cantons that have more inclusive policies: in these cantons, deaths from cardiovascular diseases, external causes, and infectious diseases are relatively less frequent among undocumented immigrants than they are in cantons with fragmented policies or with no policy. Previous research has shown that inadequate responses to the specific mortality risks of undocumented immigrants increase costs both in the health care system and society more generally [40]. Policy choices can reduce the premature mortality among undocumented immigrants, especially for those causes of death that are amenable. Understanding the association between different political choices and the mortality patterns of undocumented immigrants in different contexts remains an urgent objective for public health researchers and policy-makers.

## Supplementary Information

**Additional file 1.**

## Data Availability

The data that support the findings of this study are available from the Federal Statistical Office of Switzerland, but restrictions apply to the availability of these data, which were used under license for the current study, and so are not publicly available. Data are however available from the authors upon reasonable request and with permission of the Federal Statistical Office of Switzerland.

## Abbreviations

EFTA: European Free Trade Association
EU: European Union
FSO: Federal Statistical Office
NGO: Non-governmental organisation
WHO: World Health Organization

## References

[1] De VE, de Waure C, Specchia ML, Ricciardi W (2015). Public health aspects of migrant health: a review of the evidence on health status for undocumented migrants in the European region.

[2] Castañeda H, Holmes SM, Madrigal DS, Young M-ED, Beyeler N, Quesada J (2015). Immigration as a social determinant of health. Annu Rev Public Health.

[3] Biswas D, Kristiansen M, Krasnik A, Norredam M (2011). Access to healthcare and alternative health-seeking strategies among undocumented migrants in Denmark. BMC Public.

[4] Lebano A, Hamed S, Bradby H, Gil-Salmerón A, Durá-Ferrandis E, Garcés-Ferrer J, et al. Migrants’ and refugees’ health status and healthcare in Europe: a scoping literature review. BMC Public Health. 2020;20(1):1–22. 10.1186/s12889-020-08749-8 PMC7329528 32605605

[5] Kuehne A, Huschke S, Bullinger M (2015). Subjective health of undocumented migrants in Germany - a mixed methods approach health behavior, health promotion and society. BMC Public Health.

[6] Spitzer Denise, Torres Sara, Zwi Anthony, Khalema Ernest, Palaganas Erlinda (2019). Towards inclusive migrant healthcare. BMJ.

[7] Lockwood D (2009). Civic integration and class formation. Br J Sociol.

[8] Morris L (2003). Managing contradiction: civic stratification and migrants’ rights. Int Migr Rev.

[9] Torres JM, Waldinger R (2015). Civic stratification and the exclusion of undocumented immigrants from cross-border health care. J Health Soc Behav.

[10] Joseph TD (2017). Falling through the coverage cracks: how documentation status minimizes immigrants’ access to health care. J Health Polit Policy Law.

[11] Wallace SP, Young MEDT, Rodríguez MA, Brindis CD (2019). A social determinants framework identifying state-level immigrant policies and their influence on health. SSM Popul Health.

[12] Sweileh WM, Wickramage K, Pottie K, Hui C, Roberts B, Sawalha AF (2018). Bibliometric analysis of global migration health research in peer-reviewed literature (2000-2016). BMC Public Health.

[13] Gelatt J. Immigrant workers: vital to the U.S. COVID-19 response, disproportionately vulnerable. Migr Policy Inst. 2020;(April 2020):1–14. Available from: https://www.migrationpolicy.org/research/immigrant-workers-us-covid-19-response.

[14] Vilog RBT, Piocos CM (2021). Undocumented in the time of pandemic: exploring legal violence, health care and human rights of irregular Filipino migrants in Italy and the UK. Int J Hum Rights Healthc.

[15] Fasani F, Mazza J. Immigrant key workers: their contribution to Europe’s COVID-19 response. IZA DP. 2020;13178:1–32. Available from: http://ftp.iza.org/pp155.pdf.

[16] Woodward A, Howard N, Wolffers I (2014). Health and access to care for undocumented migrants living in the European Union: a scoping review. Health Policy Plan.

[17] Burton-Jeangros C, Duvoisin A, Lachat S, Consoli L, Fakhoury J, Jackson Y (2020). The impact of the Covid-19 pandemic and the lockdown on the health and living conditions of undocumented migrants and migrants undergoing legal status regularization. Front Public Health.

[18] Hacker K, Anies M, Folb BL, Zallman L (2015). Barriers to health care for undocumented immigrants: a literature review. Risk Manag Healthc Policy.

[19] Tjukanov N, Tiittala P, Salmi H. Health service use and costs among migrants in an irregular situation: cross-sectional register-based study from a voluntary-based clinic. J Public Health. 2021:1–4. 10.1093/pubmed/fdab382 PMC10017079 34719721

[20] Seidler Y, Novak-Zezula S, Trummer U (2019). ‘Falling off the radar’ of public health: the case of uninsured Chinese patients in Vienna, Austria. Health policy.

[21] Vatter A, Ruefli C (2003). Do political factors matter for health care expenditure? A comparative study of Swiss cantons. J Public Policy.

[22] Lijphart A. The value of within-nation comparative analysis. In: Vatter A, editor. Kantonale demokratien im vergleich opladen: Leske and Budrich. Opladen: Leske and Budrich; 2002. p. 13–15.

[23] Clandestino Project (2009). Undocumented migration: counting the uncountable. Data and trends across Europe, final report.

[24] Connor BYP, Passel JS. Europe’s Unathorized immigrant population peaks in 2016, then levels off. Pew Res Cent. 2019;November: 1–52. Available from: https://www.pewresearch.org/global/2019/11/13/europes-unauthorized-immigrantpopulation-peaks-in-2016-then-levels-off/.

[25] Morlok M, Oswald A, Meier H, Efionayi-mäder D, Ruedin D, Bader D, et al. Les sans-papiers en Suisse en 2015. Basel: BSS Volkswirtschaftliche Beratung; 2015.

[26] Spencer S, Hughes V (2015). Outside and In: legal entitlments to health care and education for migrants with irregular status in Europe.

[27] Wyssmüller C, Efionayi-Mäder D (2011). Undocumented migrants: their needs and strategies for accessing health care in Switzerland.

[28] Bilger V, Hollomey C, Wyssmüller C, Efionayi-Mäder D (2011). Health Care for Undocumented Migrants in Switzerland: Policies – People – Practices.

[29] Piccoli L (2020). Traditions of regional citizenship : explaining subnational variation of the right to healthcare for undocumented immigrants. Reg Stud.

[30] Mikkelsen L, Iburg KM, Adair T, Fürst T, Hegnauer M, von der Lippe E (2020). Assessing the quality of cause of death data in six high-income countries: Australia, Canada, Denmark, Germany, Japan and Switzerland. Int J Public Health.

[31] Wahlberg A, Källestål C, Lundgren AC, Essén B (2014). Causes of death among undocumented migrants in Sweden, 1997-2010. Glob Health Action.

[32] Lafaut D, Vandenheede H, Surkyn J, Coene G (2019). Counting the non-existing : causes of death of undocumented migrants in Brussels- Capital Region ( Belgium ), 2005–2010. Arch Public Heal.

[33] Cox DR, Snell EJ (1989). Analysis of binary data.

[34] OECD and Eurostat. Avoidable mortality: OECD/Eurostat lists of preventable and treatable causes of death (October 2021 version). 2021. Available from: https://www.oecd.org/health/health-systems/Avoidable-mortality-2019-Joint-OECD-Eurostat-List-preventable-treatable-causes-of-death.pdf.

[35] Wanner P. Inégalités spatiales de mortalité en Suisse. Le rôle de la migration internationale sur l’espérance de vie de la population des métropoles. Espac Popul sociétés. 2018;(1–2):1–24.

[36] Cheong AR, Massey DS (2019). Undocumented and unwell: legal status and health among Mexican migrants. Int Migr Rev.

[37] Holmes SM, Castañeda E, Geeraert J, Castaneda H, Probst U, Zeldes N, et al. Deservingness: migration and health in social context. BMJ Glob Health. 2021;6(1):1–5. 10.1136/bmjgh-2021-005107 PMC8031028 33827795

[38] Kickbusch I (2015). The political determinants of health - 10 years on: public health professionals need to become more politically astute to achieve their goals. BMJ.

[39] Mackenbach JP (2013). Political determinants of health. Eur J Pub Health.

[40] Ingleby D, Petrova-Benedict R (2016). Recommendations on access to health services for migrants in an irregular situation: an expert consensus.

