# Supplementary material for: The political determinants of the health of undocumented immigrants: a comparative analysis of mortality patterns in Switzerland
Source: BMC Public Health. 2022 Apr 22;22:804. doi: 10.1186/s12889-022-13188-8 (PMC9024067; doi:10.1186/s12889-022-13188-8)
Supplement: Supplementary file 1 — Additional file 1. [file 12889_2022_13188_MOESM1_ESM.docx]

**The political** determinants **of the health of undocumented immigrants: A** comparative **analysis of mortality patterns in** Switzerland

**Additional figures and tables**

Figure A1. Distribution of the deaths according to nationality status, sex, and age cohort

Source: FSO.

*Figure A2. Distribution of the age of death per legal status across the three groups of cantons*

Table A1. Deaths recorded in Switzerland, 2011 – 2017

| Basic information | |  |  |  |  |  |  |  |  |
| --- | --- | --- | --- | --- | --- | --- | --- | --- | --- |
|  |  |  | 2011 | 2012 | 2013 | 2014 | 2015 | 2016 | 2017 |
|  |  |  | N | N | N | N | N | N | N |
| Status | Nationality | Information on cause of death |  |  |  |  |  |  |  |
| Permanent resident population | Swiss | No | 1,059 | 1,141 | 1,197 | 991 | 979 | 959 | 781 |
|  |  | Yes | 55,217 | 56,926 | 57,321 | 56,448 | 59,759 | 57,189 | 58,988 |
|  | Foreigner | No | 94 | 130 | 139 | 117 | 127 | 125 | 181 |
|  |  | Yes | 5,171 | 5,399 | 5,767 | 5,754 | 6,146 | 6,043 | 6,322 |
| Non-permanent resident population | Foreigner | No | 4 | 4 | 4 | 0 | 1 | 3 | 4 |
|  |  | Yes | 109 | 156 | 64 | 51 | 64 | 71 | 58 |
| Non-resident population | Foreigner | No | 14 | 16 | 23 | 28 | 26 | 14 | 30 |
|  |  | Yes | 855 | 891 | 904 | 915 | 1,038 | 976 | 987 |

Source: FSO.

Table A2: Mean age of death and standard error, according to status, cause of death, and sex

|  | Swiss citizens | | | | Documented immigrants | | | | Undocumented immigrants | | | |
| --- | --- | --- | --- | --- | --- | --- | --- | --- | --- | --- | --- | --- |
|  | Men | | Women | | Men | | Women | | Men | | Women | |
|  | Mean | Std | Mean | Std | Mean | Std | Mean | Std | Mean | Std | Mean | Std |
| Infectious diseases | 75 | 16 | 82 | 13 | 67 | 17 | 72 | 20 | 49 | 17 | 48 | 21 |
| Neoplasms | 74 | 12 | 75 | 13 | 69 | 13 | 68 | 15 | 56 | 21 | 62 | 14 |
| Cardiovascular diseases | 81 | 12 | 87 | 9 | 74 | 14 | 82 | 12 | 62 | 16 | 67 | 14 |
| External causes | 66 | 21 | 77 | 18 | 53 | 21 | 63 | 24 | 36 | 16 | 47 | 17 |
| Other causes | 78 | 16 | 84 | 13 | 69 | 22 | 75 | 23 | 49 | 26 | 53 | 30 |
| All causes | 77 | 15 | 83 | 13 | 69 | 18 | 74 | 20 | 51 | 22 | 59 | 22 |

Source: FSO.

*Table A3 : Proportion of deaths per age cohort among Swiss citizens, documented immigrants, undocumented immigrants*

| Group of the population | Age cohort | | | | | | | | | | | | | |
| --- | --- | --- | --- | --- | --- | --- | --- | --- | --- | --- | --- | --- | --- | --- |
|  | 0-4 | 5-9 | 10-14 | 15-19 | 20-24 | 25-29 | 30-34 | 34-39 | 40-44 | 45-49 | 50-54 | 55-59 | 60-64 | 0-64 |
| Swiss citizens | 3.3% | 0.3% | 0.3% | 1.0% | 1.7% | 1.8% | 2.1% | 2.7% | 4.8% | 9.4% | 15.7% | 23.6% | 33.3% | 100% |
| Documented immigrants | 10.7% | 0.5% | 0.6% | 1.4% | 2.4% | 3.7% | 4.5% | 6.4% | 6.8% | 10.5% | 13.9% | 18.7% | 19.9% | 100% |
| Undocumented immigrants | 7.4% | 2.1% | 0.5% | 2.1% | 3.8% | 9.3% | 9.8% | 8.3% | 8.6% | 9.3% | 11.2% | 12.6% | 15.0% | 100% |

*Table A4 : Distribution of origin among deceased undocumented immigrants in the three groups of cantons*

|  | Group I (Inclusive Policies) | Group II (Fragmented Policies) | Group III (No Policies) |
| --- | --- | --- | --- |
| Asia | 22.5% | 30.7% | 21.4% |
| Other Africa | 19.4% | 7.4% | 6.8% |
| The Maghreb | 18.1% | 6.3% | 2.9% |
| Balkan countries | 15.0% | 33.2% | 36.9% |
| Latin America | 14.5% | 4.6% | 7.8% |
| Other Europe | 10.6% | 17.8% | 24.3% |
| All | 100.0% | 100.0% | 100.0% |

Source: FSO.

*Table A5. Distribution of the causes of death of Swiss citizens and documented immigrants according to the group of cantons and sex (in %)*

|  | Swiss citizens | | | Documented immigrants | | |
| --- | --- | --- | --- | --- | --- | --- |
|  | Group 1:  Inclusive Policies | Group 2:  Fragmented Policies | Group 3:  No Policy | Group 1:  Inclusive Policies | Group 2:  Fragmented Policies | Group 3: No Policy |
| Men |  |  |  |  |  |  |
| Infectious Diseases | 1.1 | 1.1 | 1.0 | 1.2 | 1.4 | 1.5 |
| Neoplasms | 28.7 | 27.7 | 27.9 | 33.5 | 33.5 | 34.4 |
| Circulatory Diseases | 25.7 | 30.0 | 31.5 | 21.8 | 23.9 | 25.2 |
| External deaths | 7.7 | 7.4 | 7.3 | 8.1 | 8.8 | 9.0 |
| Other Causes | 36.8 | 33.8 | 32.3 | 35.4 | 32.5 | 29.9 |
|  | 100.0 | 100.0 | 100.0 | 100.0 | 100.0 | 100.0 |
| Women |  |  |  |  |  |  |
| Infectious Diseases | 0.9 | 1.1 | 1.1 | 0.9 | 1.6 | 1.5 |
| Neoplasms | 21.8 | 20.9 | 20.8 | 26.3 | 31.7 | 29.9 |
| Circulatory Diseases | 28.5 | 33.5 | 34.9 | 25.5 | 25.3 | 27.9 |
| External deaths | 5.8 | 5.5 | 4.8 | 6.4 | 5.4 | 5.9 |
| Other Causes | 43.0 | 39.0 | 38.5 | 40.8 | 36.0 | 34.9 |
|  | 100.0 | 100.0 | 100.0 | 100.0 | 100.0 | 100.0 |

Source: SFSO, BEVNAT

Table A6. Correlation of status and cause of death, controlled for age and origin

|  | Undocumented immigrants versus documented immigrants of the same origin | | | | | | | |
| --- | --- | --- | --- | --- | --- | --- | --- | --- |
|  | Men | | | | Women | | | |
|  | OR | 95% CI | | P | OR | 95% CI | | P |
| Infectious diseases | 1.1 | 0.5 - 2.3 | |  | 0.8 | 0.3 - 1.9 | |  |
| Europe (ref) | 1.0 |  |  |  | 1.0 |  |  |  |
| Africa | 3.9 | 2.1 - 7.1 | | *** | 3.1 | 1.7 - 5.8 | | *** |
| Latin America | 0.5 | 0.1 - 3.9 | |  | 0.7 | 0.2 - 2.2 | |  |
| Asia | 2.4 | 1.2 - 4.5 | | ** | 1.4 | 0.7 - 2.8 | |  |
| Neoplasms | 0.5 | 0.4 - 0.7 | | *** | 0.6 | 0.4 - 0.8 | | *** |
| Europe (ref) | 1.0 |  |  |  | 1.0 |  |  |  |
| Africa | 0.8 | 0.6 - 1.0 | | * | 0.9 | 0.7 - 1.2 | |  |
| Latin America | 1.0 | 0.7 - 1.4 | |  | 0.9 | 0.6 - 1.2 | |  |
| Asia | 0.5 | 0.4 - 0.7 | | *** | 0.7 | 0.6 - 0.9 | | * |
| Cardiovascular diseases | 1.9 | 1.5 - 2.4 | | *** | 1.9 | 1.4 - 2.6 | | *** |
| Europe (ref) | 1.0 |  |  |  | 1.0 |  |  |  |
| Africa | 0.8 | 0.6 - 1.0 | | * | 0.7 | 0.5 - 1.1 | |  |
| Latin America | 0.4 | 0.3 - 0.7 | | *** | 0.8 | 0.5 - 1.1 | |  |
| Asia | 1.1 | 0.9 - 1.3 | |  | 1.2 | 1.0 - 1.6 | |  |
| External causes | 1.7 | 1.3 - 2.2 | | *** | 1.4 | 0.9 - 2.3 | |  |
| Europe (ref) | 1.0 |  |  |  | 1.0 |  |  |  |
| Africa | 1.0 | 0.8 - 1.4 | |  | 0.7 | 0.4 - 1.3 | |  |
| Latin America | 2.2 | 1.4 - 3.2 | | *** | 1.7 | 1.1 - 2.7 | |  |
| Asia | 1.2 | 0.9 - 1.5 | |  | 1.6 | 1.1 - 2.4 | | * |
| Other causes | 0.7 | 0.6 - 0.9 | | *** | 0.9 | 0.7 - 1.2 | |  |
| Europe (ref) | 1.0 |  |  |  | 1.0 |  |  |  |
| Africa | 1.4 | 1.1 - 1.7 | | * | 1.3 | 0.9 - 1.7 | |  |
| Latin America | 1.1 | 0.8 - 1.5 | |  | 1.2 | 0.9 - 1.6 | |  |
| Asia | 1.5 | 1.2 - 1.8 | | ** | 0.9 | 0.7 - 1.2 | |  |

Source: Own elaboration, based on data from FSO.

*Table A7. Odds ratios of mortality for different causes among undocumented immigrants compared to Swiss citizens, documented immigrants and documented immigrants of the same origin, unadjusted from age.*

|  | Undocumented versus Swiss | | | | | |
| --- | --- | --- | --- | --- | --- | --- |
|  | Men | | | Women | | |
|  | OR | 95% CI | P | OR | 95% CI | P |
| Infectious diseases | 2.0 | 1.0 - 3.8 | * | 2.3 | 1.0 - 5.2 | * |
| Neoplasms | 0.5 | 0.4 - 0.7 | *** | 1.2 | 0.9 - 1.6 |  |
| Cardiovascular diseases | 1.0 | 0.8 - 1.2 |  | 0.9 | 0.7 - 1.1 |  |
| External causes | 4.1 | 3.3 - 5.1 | *** | 1.8 | 1.2 - 2.7 | ** |
| Other causes | 0.7 | 0.6 - 0.9 | ** | 0.8 | 0.6 - 1.0 |  |
|  | Undocumented versus documented immigrants | | | | | |
|  | Men | | | Women | | |
|  | OR | 95% CI | P | OR | 95% CI | P |
| Infectious diseases | 1.5 | 0.8 - 3.0 |  | 1.7 | 0.8 - 3.9 |  |
| Neoplasms | 0.4 | 0.3 - 0.5 | *** | 0.8 | 0.6 - 1.0 |  |
| Cardiovascular diseases | 1.3 | 1.1 - 1.6 | * | 1.2 | 0.9 - 1.6 |  |
| External causes | 3.5 | 2.8 - 4.3 | *** | 1.6 | 1.1 - 2.5 | * |
| Other causes | 0.8 | 0.6 - 1.0 | * | 0.9 | 0.7 - 1.1 |  |
|  | Undocumented versus documented immigrants of the same origin | | | | | |
|  | Men | | | Women | | |
|  | OR | 95% CI | P | OR | 95% CI | P |
| Infectious diseases | 1.5 | 0.7 - 3.0 |  | 1.0 | 0.4 - 2.3 |  |
| Neoplasms | 0.4 | 0.3 - 0.5 | *** | 0.6 | 0.5 - 0.8 | ** |
| Cardiovascular diseases | 1.5 | 1.2 - 1.9 | *** | 1.6 | 1.2 - 2.2 | *** |
| External causes | 2.6 | 2.1 - 3.3 | *** | 1.4 | 0.9 - 2.2 |  |
| Other causes | 0.8 | 0.6 - 1.0 | * | 0.9 | 0.7 - 1.2 |  |

Source: Own elaboration, based on data from FSO. Results of logistic regressions. * p < 0.05 ** p < 0.01 *** p < 0.001.

*Table A8. Proportion of amenable and preventable deaths for status and sex*

|  | | | Men | | Women | | Both sexes | |
| --- | --- | --- | --- | --- | --- | --- | --- | --- |
|  |  |  | Preventable deaths | Amenabledeaths | Preventabledeaths | Amenable deaths | Preventable deaths | Amenable deaths |
| Swiss |  | % of all deaths within this group of the population | 22.3 | 10.0 | 14.4 | 7.1 | 18.1 | 8.5 |
|  |  | Number of deaths | 42583 | 19141 | 31460 | 15493 | 74043 | 34634 |
|  |  |  |  |  |  |  |  |  |
| Documented immigrants |  | % of all deaths within this group of the population | 31.6 | 15.0 | 21.4 | 14.5 | 27.6 | 14.8 |
|  |  | Number of deaths | 7967 | 3788 | 3483 | 2368 | 11450 | 6156 |
|  |  |  |  |  |  |  |  |  |
| Undocumented  immigrants | | % of all deaths within this group of the population | 50.2 | 27.6 | 38.6 | 35.9 | 45.9 | 30.6 |
|  |  | Number of deaths | 215 | 118 | 97 | 90 | 312 | 208 |

Source: Own elaboration based on data from FSO and definition of Eurostat/OECD.

Table A9. Correlation of status and cause of death, cantons of Group 1 (Inclusive Policies). Control: age.

|  | Undocumented immigrants versus Swiss citizens | | | | | | | |
| --- | --- | --- | --- | --- | --- | --- | --- | --- |
|  | Men | | | | Women | | | |
|  | OR | 95% CI | | P | OR | 95% CI | | P |
| Infectious diseases | 1.0 | 0.2 - 4.2 | |  | no case |  |  |  |
| Neoplasms | 0.8 | 0.5 - 1.2 | |  | 0.6 | 0.4 - 1.0 | |  |
| Cardiovascular diseases | 1.8 | 1.2 - 2.7 | | ** | 2.1 | 1.2 - 3.6 | | ** |
| External causes | 0.5 | 0.3 - 0.9 | |  | 0.3 | 0.1 - 0.9 | | * |
| Other causes | 1.2 | 0.9 - 1.7 | |  | 1.5 | 0.9 - 2.4 | |  |
|  | Undocumented versus documented immigrants | | | | | | | |
|  | Men | | | | Women | | | |
|  | OR | 95% CI | | P | OR | 95% CI | | P |
| Infectious diseases | 1.0 | 0.2 - 4.1 | |  | no case |  |  |  |
| Neoplasms | 0.6 | 0.4 - 1.0 | | * | 0.6 | 0.3 - 1.0 | |  |
| Cardiovascular diseases | 1.8 | 1.1 - 2.7 | | ** | 1.8 | 1.0 - 3.2 | | * |
| External causes | 0.7 | 0.4 - 1.1 | |  | 0.5 | 0.2 - 1.3 | |  |
| Other causes | 1.2 | 0.9 - 1.8 | |  | 1.4 | 0.9 - 2.4 | |  |
|  | Undocumented versus documented immigrants of the same origin | | | | | | | |
|  | Men | | | | Women | | | |
|  | OR | 95% CI | | P | OR | 95% CI | | P |
| Infectious diseases | 0.9 | 0.2 - 4.2 | |  | no case |  |  |  |
| Neoplasms | 0.7 | 0.4 - 1.1 | |  | 0.7 | 0.4 - 1.2 | |  |
| Cardiovascular diseases | 1.5 | 1.0 - 2.5 | |  | 1.5 | 0.8 - 2.7 | |  |
| External causes | 0.9 | 0.5 - 1.7 | |  | 0.6 | 0.2 - 1.8 | |  |
| Other causes | 1.0 | 0.7 - 1.6 | |  | 1.4 | 0.8 - 2.4 | |  |
| Source: FSO. Own elaboration. |  |  |  |  |  |  |  |  |

Table A10. Correlation of status and cause of death, cantons of Group 2 (Fragmented Policies). Control: age.

|  | Undocumented immigrants versus Swiss citizens | | | | | | | |
| --- | --- | --- | --- | --- | --- | --- | --- | --- |
|  | Men | | | | Women | | | |
|  | OR | 95% CI | | P | OR | 95% CI | | P |
| Infectious diseases | 1.9 | 0.8 - 4.6 | |  | 3.6 | 1.5 - 8.7 | | ** |
| Neoplasms | 0.5 | 0.4 - 0.7 | | *** | 0.5 | 0.4 - 0.8 | | ** |
| Cardiovascular diseases | 2.4 | 1.8 - 3.2 | | *** | 2.6 | 1.8 - 3.8 | | *** |
| External causes | 1.6 | 1.1 - 2.2 | | * | 1.2 | 0.7 - 2.1 | |  |
| Other causes | 0.5 | 0.4 - 0.7 | | *** | 0.7 | 0.4 - 1.0 | | * |
|  | Undocumented versus documented immigrants | | | | | | | |
|  | Men | | | | Women | | | |
|  | OR | 95% CI | | P | OR | 95% CI | | P |
| Infectious diseases | 1.6 | 0.7 - 4.0 | |  | 2.1 | 0.8 - 5.3 | |  |
| Neoplasms | 0.4 | 0.3 - 0.6 | | *** | 0.5 | 0.3 - 0.7 | | *** |
| Cardiovascular diseases | 2.3 | 1.7 - 3.1 | | *** | 2.3 | 1.6 - 3.4 | | *** |
| External causes | 1.9 | 1.3 - 2.8 | | *** | 1.9 | 1.1 - 3.4 | | * |
| Other causes | 0.5 | 0.4 - 0.8 | | *** | 0.7 | 0.5 - 1.0 | |  |
|  | Undocumented versus documented immigrants of the same origin | | | | | | | |
|  | Men | | | | Women | | | |
|  | OR | 95% CI | | P | OR | 95% CI | | P |
| Infectious diseases | 1.4 | 0.5 - 3.7 | |  | 1.3 | 0.5 - 3.5 | |  |
| Neoplasms | 0.4 | 0.3 - 0.6 | | *** | 0.6 | 0.4 - 0.9 | | *** |
| Cardiovascular diseases | 2.0 | 1.5 - 2.8 | | *** | 2.0 | 1.3 - 3.0 | | *** |
| External causes | 2.4 | 1.6 - 3.5 | | *** | 2.4 | 1.3 - 4.4 | | ** |
| Other causes | 0.6 | 0.4 - 0.8 | | ** | 0.6 | 0.4 - 1.0 | | * |
| Source: FSO. Own elaboration. |  |  |  |  |  |  |  |  |

Table A11. Correlation of status and cause of death, cantons of Group 3 (No policy). Control: age.

|  | Undocumented immigrants versus Swiss citizens | | | | | | | |
| --- | --- | --- | --- | --- | --- | --- | --- | --- |
|  | Men | | | | Women | | | |
|  | OR | 95% CI | | P | OR | 95% CI | | P |
| Infectious diseases | 3.2 | 0.8 - 13.5 | |  | 2.2 | 0.3 - 16.5 | |  |
| Neoplasms | 0.3 | 0.1 - 0.6 | | *** | 0.2 | 0.1 - 0.6 | | ** |
| Cardiovascular diseases | 2.4 | 1.4 - 4.1 | | ** | 2.5 | 1.2 - 5.3 | | * |
| External causes | 1.1 | 0.6 - 2.1 | |  | 0.9 | 0.3 - 3.1 | |  |
| Other causes | 0.9 | 0.5 - 1.6 | |  | 1.4 | 0.7 - 2.8 | |  |
|  | Undocumented versus documented immigrants | | | | | | | |
|  | Men | | | | Women | | | |
|  | OR | 95% CI | | P | OR | 95% CI | | P |
| Infectious diseases | 1.8 | 0.4 - 7.9 | |  | 1.9 | 0.3 - 14.5 | |  |
| Neoplasms | 0.2 | 0.1 - 0.5 | | *** | 0.2 | 0.1 - 0.6 | | ** |
| Cardiovascular diseases | 2.3 | 1.3 - 3.9 | | ** | 2.4 | 1.2 - 5.2 | | * |
| External causes | 1.3 | 0.7 - 2.5 | |  | 1.0 | 0.3 - 3.7 | |  |
| Other causes | 1.0 | 0.6 - 1.7 | |  | 1.6 | 0.8 - 3.3 | |  |
|  | Undocumented versus documented immigrants of the same origin | | | | | | | |
|  | Men | | | | Women | | | |
|  | OR | 95% CI | | P | OR | 95% CI | | P |
| Infectious diseases | 2.6 | 0.5 - 13.0 | |  | 0.9 | 0.1 - 7.3 | |  |
| Neoplasms | 0.2 | 0.1 - 0.5 | | *** | 0.2 | 0.1 - 0.6 | | ** |
| Cardiovascular diseases | 2.1 | 1.2 - 3.7 | | * | 2.5 | 1.2 - 5.5 | | * |
| External causes | 2.3 | 1.2 - 4.5 | | * | 1.4 | 0.4 - 5.9 | |  |
| Other causes | 0.8 | 0.4 - 1.4 | |  | 1.4 | 0.7 - 3.1 | |  |
| Source: FSO. Own elaboration. |  |  |  |  |  |  |  |  |
